# Dr. Robert Southgate

**Published:** 1864-04

**Authors:** 


### *Obituary.*

Amid the sanguinary scenes which so often attend the birth of a new nation; where peaceful, happy homes are daily lost, and human life balances on the slenderest thread, the death of an individual is ordinarily but little felt or regarded. In the decease, however, of Dr. ROBERT SOUTHGATE, the profession, of which he was a brilliant member, has sustained a serious loss, and a large circle of admiring friends a severe affliction.

In his earlier years, Dr. Southgate was connected with the medical staff of the United States army, from which he resigned some time before the commencement of the war to engage in civil practice.

Having established himself at St. Augustine, Florida, he was there actively employed in professional pursuits, when Virginia, his native State, seceded. At once he abandoned home and interest, and tendered to her governor his services, which were immediately accepted. Passing from the State service shortly after to the Provisional Army of the Confederate States, he only ceased to serve them diligently and faithfully with his death, which occurred on the evening of the 23d instant.

Dr. Southgate was conspicuous, in and out of the army, not alone for his richly stored mind, and his rare and brilliant talents, but for a mild and gentle character, and a pure, spotless morality. In his demise, his afflicted family, his professional brethren, the army and the country have, indeed, sustained an irreparable loss. Peace to his ashes.

